# Mapping the “X” Debate: Water Fluoridation Sentiment Analysis With Advanced Machine Learning

**DOI:** 10.1111/jphd.12669

**Published:** 2025-05-07

**Authors:** Nilesh Torwane, Ratilal Lalloo, Diep Ha, Loc Do

**Affiliations:** ^1^ School of Dentistry University of Queensland Herston Australia

**Keywords:** community water fluoridation, machine learning, public health, sentiment analysis, social media

## Abstract

**Objectives:**

This study aimed to examine public sentiment regarding community water fluoridation (CWF) using data from “X” (formerly Twitter) over the past decade. The goal was to understand public opinion trends and identify opportunities for targeted public health communication.

**Methods:**

We conducted a sentiment analysis utilizing a natural language processing technique. Specifically, we applied the Sentiment Intensity Analyzer tool to classify tweets related to CWF into negative, positive, or neutral categories. Additionally, a word co‐occurrence network analysis was performed to explore key discussion themes. We also compared machine learning models to assess their accuracy in classifying tweet sentiments.

**Results:**

Analysis of the tweets revealed a balanced distribution of sentiments: 37.4% negative, 34.4% positive, and 28.2% neutral. Peaks in public engagement occurred between 2015 and 2016, with a subsequent decline after 2018. Sentiment spikes were often associated with significant events, including policy changes and media coverage. The word co‐occurrence network highlighted recurring themes related to safety and dental health. Among the machine learning models evaluated, Logistic Regression demonstrated the highest accuracy in sentiment classification.

**Conclusions:**

Our findings highlight the polarized nature of public sentiment toward CWF and the temporal fluctuations in public engagement. These insights can inform public health policymakers in developing timely, targeted communication strategies. Specifically, efforts to engage neutral audiences through transparent messaging and counter misinformation during key periods may strengthen public trust in CWF. The application of machine learning in this analysis underscores its value in enhancing real‐time monitoring and supporting evidence‐based public health strategies.

## 1 Introduction

Social networking services (SNSs) such as “X” (formerly Twitter), Facebook, and Instagram significantly influence public perceptions by acting as both sources of information and breeding grounds for mis(dis)information [1]. The term *“mis(dis)information”* encompasses both *misinformation*—unintentional inaccuracies or misleading statements—and *disinformation*—deliberate falsehoods created to manipulate public opinion. Recognizing this duality underscores how both phenomena can simultaneously shape online discussions and public perceptions [1, 2]. This capability extends across political, scientific, technological, and social arenas, swaying public opinion in diverse ways [2].

SNSs play a crucial role in public discussions on numerous health‐related issues, such as vaccine debates, drug safety, and chronic non‐communicable diseases, impacting individuals' decision‐making abilities [1, 2, 3] and have contributed to public health crises. Instances of false or misleading information on vaccines on social media platforms such as Twitter and Facebook have been associated with a reluctance to be vaccinated, resulting in epidemics of measles in different countries [1, 2]. Similarly, the dissemination of inaccurate information on the safety of drugs has resulted in alterations in medicine usage, which has had an adverse impact on public health outcomes [3, 4]. Although SNSs cannot directly cause worldwide epidemics, they have the potential to magnify mis(dis)information. This can worsen public health problems by quickly impacting many people.

Community water fluoridation (CWF) is one public health intervention extensively impacted by mis(dis)information [5, 6]. For example, some SNS discussions inaccurately link fluoride to severe health issues such as thyroid dysfunction, bone disorders, or decreased IQ, or promote conspiracy theories that undermine the scientific consensus, despite substantial evidence supporting the safety and effectiveness of CWF [5, 6, 7]. Over eight decades of research have consistently demonstrated that CWF is a safe and cost‐effective method for reducing dental caries across populations, with notable benefits in both children and adults [7, 8, 9]. The World Health Organisation (WHO) and the centers for disease control and prevention (CDC) have recognized CWF as one of the most significant public health achievements of the 20th century, citing its ability to reduce dental decay by approximately 25% in communities with fluoridated water [8, 9, 10]. These endorsements underscore the robust scientific consensus supporting CWF as a critical measure for improving oral health outcomes.

Despite this, public sentiments on CWF remain diverse, inconsistent, and poorly understood [8, 9]. The internet, particularly SNSs, presents CWF negatively due to publicized outlier research [5, 6]. These digital trends, if not addressed, can significantly impact policy decisions and alter the course of this essential public health initiative [11, 12, 13]. This emphasizes the urgent need for real‐time monitoring of public opinion on CWF due to the rapid and frequent shifts in public attitudes and perceptions. To make informed decisions on CWF, dental practitioners, health policymakers, and government bodies must understand public sentiment [11, 12]. Among social networking sites, “X” stands out due to its widespread use, with over 300 million monthly users sharing and receiving information; this SNS gives researchers unique insights into the topic that might be more difficult to obtain through standard surveys [3, 4]. “X” is a valuable source of information for studying public opinions and evaluating mis(dis)information about health‐related topics such as immunizations and nutrition guidelines, leading to more effective and informed public health interventions [2, 3, 4]. However, the dentistry sector has yet to fully realize the potential of “X” data for research [5, 6, 11]. Regarding CWF, there has been only one study by Oh et al. [11] using “X” to analyze public sentiment. This study identified public opinion trends but did not focus on the main positive and negative elements of the conversations. It also did not conduct a detailed network analysis of common themes or how different types of tweets influence public sentiment. Additionally, the study did not compare the accuracy of various machine learning models, and its data are outdated, making it less relevant given the fast‐changing nature of online discussions.

Several historical events further underscore the relevance of studying CWF sentiment on “X.” For example, in 2015, the United States Department of Health and Human Services updated its fluoridation guidelines, reducing the recommended level to minimize fluorosis, sparking debates on platforms like “X” [12]. In 2016, cities like Calgary (Canada) and Christchurch (New Zealand) discontinued fluoridation in certain areas, generating widespread online discussions [14]. During the COVID‐19 pandemic (2020–2021), public health priorities shifted, temporarily reducing attention to issues like CWF, although online debates about its relevance persisted [15]. In 2021, debates intensified in the United Kingdom regarding plans to expand CWF coverage [5]. In Brazil, the 2013 revocation of its water fluoridation law ignited significant public discourse, much of it reflected on “X” [11, 12]. These examples illustrate how social media platforms have been instrumental in framing and influencing public sentiment regarding CWF over time.

To address the identified gaps, this study comprehensively analyzed public sentiment regarding CWF as expressed on “X.” Our primary objective was to explore how public opinions on CWF were formed and influenced by various factors, including significant events and policy changes. We aimed to identify and analyze the distribution of negative, positive, and neutral sentiments across different periods and events, revealing the dominant themes and concerns expressed by the public. Furthermore, the study sought to evaluate the effectiveness of various machine learning algorithms in accurately classifying these sentiments, thereby offering insights that could guide future research and improve analytical approaches in this field. Ultimately, our research aimed to enhance public health communication and inform CWF policy by providing an up‐to‐date understanding of global social media perceptions, thereby supporting more informed and effective public health strategies.

## 2 Material and Methods

### 2.1 Scope of the Study

The sentiment analysis was conducted using a dataset from “X.” Our research covered the period from 2014 to 2023 and is based on various parameters, including textual sentiment analysis, keyword analysis, topic modeling, and model performance testing, all relevant to CWF. These 10 years were chosen to capture a comprehensive view of sentiment trends and public discussions over a significant period, allowing us to identify long‐term patterns and shifts in public opinion. Additionally, this timeframe builds upon the methodologies recommended in prior research [3, 4, 11, 12, 13], ensuring a systematic and rigorous assessment aligned with established standards.

### 2.2 Data Confidentiality and Ethical Considerations

The sentiment analysis did not include human participants and utilized only publicly available data. Exemption from ethical review was granted by the Institute's Human Ethics Committee (Project Number: 2022/HE002248).

To ensure the anonymity of participants, we have adopted several processes. At first, all datasets were password‐encrypted while stored to prevent unauthorized access. The personally identifiable information (PII), such as identities, was anonymized through unique identifiers as substitutes. The software utilized for data extraction automatically performed the anonymization procedure, guaranteeing that personal identity details were concealed from the researchers. The investigators were granted exclusive access to the data, ensuring the safeguarding of critical information. In addition, we adhered to data minimization standards by gathering just the essential data for analysis and removing or obscuring any irrelevant PII. Our data processing system executed all these processes to guarantee thorough data security and participant privacy.

### 2.3 Sentiment Analysis Stages

**FIGURE 1:**
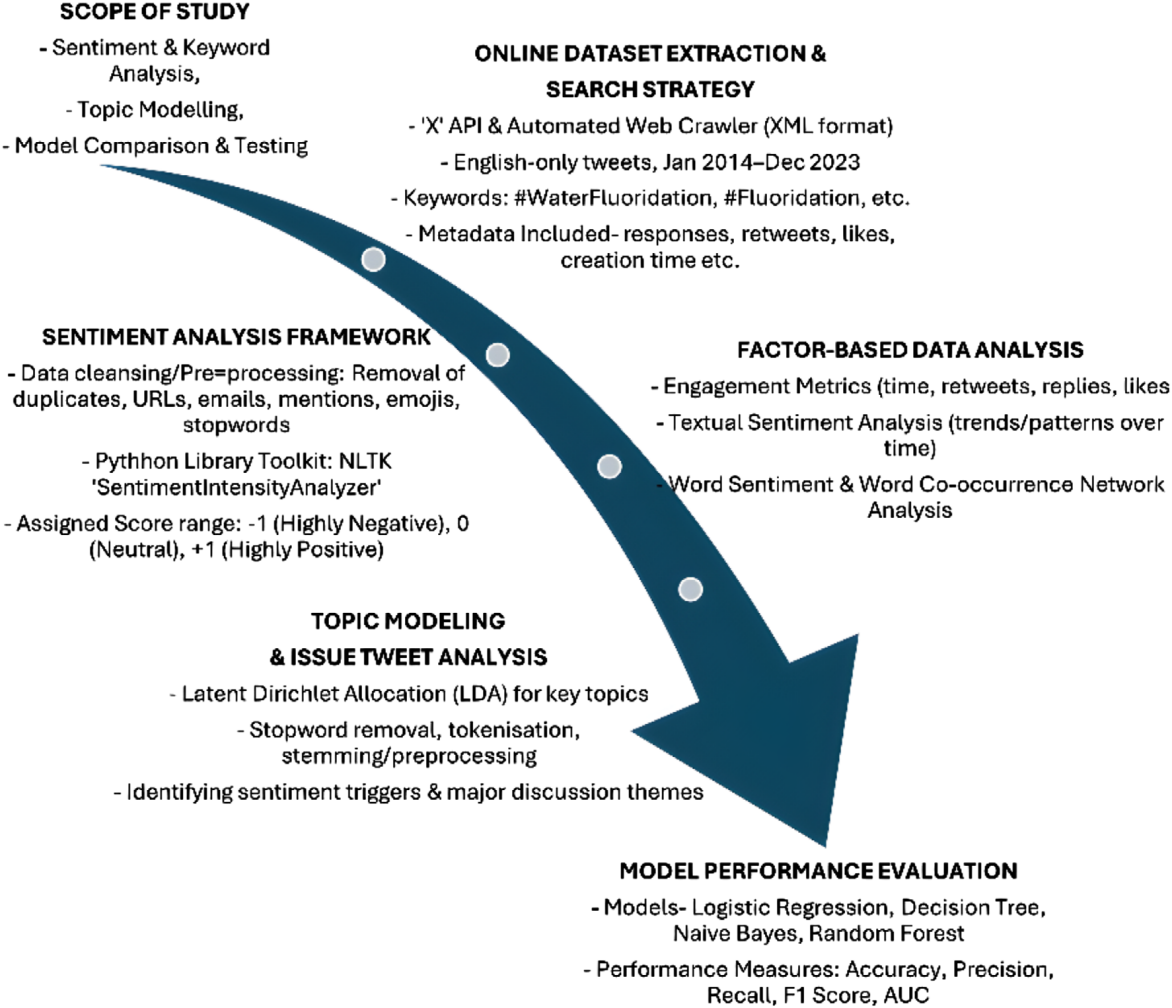
Analytical workflow for CWF sentiment analysis. This flowchart illustrates the complete analytical workflow for examining public sentiment on CWF on “X.” The process begins with dataset extraction and preprocessing, followed by sentiment analysis, topic modeling, and factor‐based data analysis. The final step involves model performance evaluation using various machine learning classifiers (e.g., Logistic Regression, Naïve Bayes, Random Forest) and metrics such as accuracy, precision, recall, and F1‐score. This structured approach ensures comprehensive insight into public discourse on CW*F*. [Color figure can be viewed at wileyonlinelibrary.com]

The process of sentimental analysis involved the following stages (Figure 1).

#### 2.3.1 SSOnline Dataset Extraction

Public sentiment regarding CWF was assessed as consistent with previous research on vaccination hesitancy [2, 15, 16]. Utilizing Python libraries and “X”'s open Application Programming Interface (API), data from January 2014 to December 2023 were obtained from “X.” The data was captured and transferred into XML format by an automated web crawler.

*To ensure accuracy and relevance in the dataset, we implemented the following measures*:

1. Keyword based filtering: Only tweets containing the specified hashtags and keywords were retained thereby minimizing off‐topic content
2. Language consistency: Non‐English tweets were excluded, as standardized linguistic tools are predominantly designed for English.
3. Topical relevance check: Tweets flagged as purely promotional or evidently off‐topic (e.g., referencing fluoride outside dental/public health contexts) were filtered out through manual inspection of a random subset and by performing Topic Modeling to identify common topics that emerge within collected tweets [11].
4. Duplicate removal: Identical or near‐duplicate tweets (e.g., retweets without additional commentary) were consolidated to prevent skewed outcomes [3, 4].

These steps helped ensure that the final dataset accurately reflected genuine discussions around CWF, aligning with our research aims. Metadata elements, including the number of responses, mentions, retweets, creation time, and favorites, were included in the collected data to provide a deeper analysis of user engagement and interactions.

#### 2.3.2 Data Source and Search Strategy

An open API collected data from “X” [11, 13, 15]. The API facilitated the collection of tweets that adhered to the specified search criteria and granted access to metadata, including user IDs, mentions, creation time, responses, retweets, and likes. This comprehensive approach guaranteed that our dataset effectively captured diverse discussions regarding CWF. The search was conducted using the following keywords: “#WaterFluoridation,” “#CommunityWaterFluoridation,” “#Fluoridation,” “#Fluoridatedwater,” and “#FluorideInWater.”

#### 2.3.3 Sentiment Analysis Framework

We implemented a structured sentiment analysis methodology to understand the public's reactions and opinions about CWF. The collected datasets were subjected to a thorough cleansing process using Python libraries involving identifying information such as URLs, emails, user mentions, punctuation, stopwords, emojis, and emoticons. This process was completed to convert the text into a standardized format suitable for sentiment analysis. The “SentimentIntensityAnalyzer” from the Natural Language Processing Toolkit (NLTK) library was employed to execute fundamental Natural Language Processing (NLP) operations for the sentiment analysis. The NLTK framework enabled us to tokenize text, divide sentences, tag parts of speech, and recognize named entities, thereby establishing a comprehensive modular pipeline for NLP tasks [17, 18, 19].

The ‘SentimentIntensityAnalyzer’ from NLTK is effective for data analysis due to its efficacy in handling short text, typical of social media platforms such as “X,” and its simplicity and reliability, compared to other models like TextBlob, Stanford NLP, Spacy, and SentiStrength. The SentimentIntensityAnalyzer is intended to comprehend the context of words within a sentence and generate a sentiment score [19]. It provides a sentiment score that accurately depicts the intensity of sentiment and effectively differentiates between positive, negative, and neutral sentiments. This renders it optimal for analyzing the informal and context‐rich language frequently present on social media. Additionally, NLTK is a versatile, well‐documented, and widely used toolset, rendering it a dependable option for our sentiment analysis requirements [18, 19, 20].

The sentiment scores generated by NLTK ranged between the following values: 1 (*Highly Positive*), 0 (*Neutral*), and −1 (*Highly Negative*) (Data S1). This allowed us to quantify the emotional tone of each tweet. The NLTK architecture was instrumental in extracting meaningful sentiment data from tweets, which was essential for our analysis.

#### 2.3.4 Factor‐Based Data Analysis

##### 2.3.4.1 Engagement Metrics Analyses

We implemented algorithms to analyze data, including the time tweets were generated and the quantity of responses, retweets, and likes they received [11]. These metrics enabled us to determine the percentage of “X” users who have discussed CWF over time, revealing interest trends. Furthermore, we analyzed fluctuations in this proportion to ascertain whether public engagement with CWF topics had increased or decreased. The trends of public engagement were further investigated by monitoring the rise and decline of related tweets, retweets, and likes.

##### 2.3.4.2 Textual Sentiment Analysis

This includes sentiment trends and patterns analysis, word sentiment analysis, and word co‐occurrence network analysis. Sentiment Trends and Patterns:

The general sentiment trends and pattern changes were monitored to comprehend the consensus among “X” users regarding CWF and its evolution over time. This analysis illustrated fluctuations in sentiment in response to critical events or discussions and provided insights into whether public opinion was predominantly positive, negative, or neutral at various points. By monitoring these trends, we were able to gain a comprehensive understanding of the changing public sentiment regarding CWF [11, 12, 13].

##### 2.3.4.3 Word Sentiment Analysis

We analyzed word sentiment to identify tweets with the most frequently used positive and negative terms. A lexicon of words with quantitative emotion scores that were pre‐assigned was employed. The sentiment scores for terms in each tweet were analyzed after removing stopwords [4, 11]. The formula was used to determine the normalized frequency of terms for each year:

$\text{Normalised frequency}=\frac{\text{Number of instances of}\;a\;\text{term in}\;a\;\text{particular year}}{\text{Number of tweets sent in that year}}$

##### 2.3.4.4 Word Co‐Occurrence Network Analysis

We established a network of co‐occurring terms by examining the most frequently used keywords. This analysis enhanced our understanding of the connections between tweet topics and key terms [11, 12, 13, 20].

##### 2.3.4.5 Topic Modeling and Issue Tweet Analysis

We analyzed tweets that addressed specific issues to explore the most popular topics “X” users were interested in each year. Furthermore, topic modeling was implemented to classify emotions and pinpoint problems that triggered them during sentiment surges [11, 12, 13, 15, 20]. We implemented the Latent Dirichlet Allocation (LDA) model [21], a prevalent topic‐modeling methodology to identify pertinent issues and extract concise, specific, and meaningful topics. This approach involved iterative sampling and word shuffling. Data preprocessing phases were implemented to prepare the text for analysis, including stopword removal, tokenization, and stemming [2, 11]. This all‐encompassing methodology enabled us to investigate the fundamental themes and sentiments expressed in the tweets, thereby offering a deeper understanding of the public discussions regarding CWF.

##### 2.3.4.6 Evaluation of Model Performance

The sentiment analysis of CWF conversations on “X” was conducted using the following models: Logistic Regression, Decision Tree, Naive Bayes, and Random Forest. These models are well‐established for their effectiveness and are frequently employed in text classification tasks. Standard machine learning performance metrics, such as accuracy, precision, recall, F1 score, and area under the curve (AUC), were employed to evaluate the classification capabilities of these models [16].

1. *Accuracy* measures the proportion of correctly classified instances out of the total cases.
2. *Precision* indicates the proportion of true positive predictions among all positive predictions, reflecting the accuracy of the positive predictions.
3. *Recall* measures the proportion of true positive predictions among all actual positives, indicating the model's ability to identify all relevant instances.
4. *F1 Score* is the harmonic mean of precision and recall, providing a metric that balances both concerns.
5. *Area under the curve (AUC)* represents the ability of the model to distinguish between classes, with a higher AUC indicating better model performance.

##### 2.3.4.7 Hyperparameter Tuning

We applied the following approach to hyperparameter tuning:

- Decision Tree and Random Forest: A grid search procedure was combined with k‐fold cross‐validation to optimize parameters such as maximum tree depth, minimum samples required for a split, and the number of estimators in the Random Forest [16, 18].
- Logistic Regression and Naive Bayes: Except for selecting an appropriate solver in Logistic Regression, we primarily retained the default settings for these models due to their established performance in text classification tasks [16, 18, 19, 20].

This balanced strategy allowed for both computational feasibility and performance refinement, thereby enhancing the transparency and credibility of our results.

## 3 Results

### 3.1 Engagement Metrics Analyses

**FIGURE 2:**
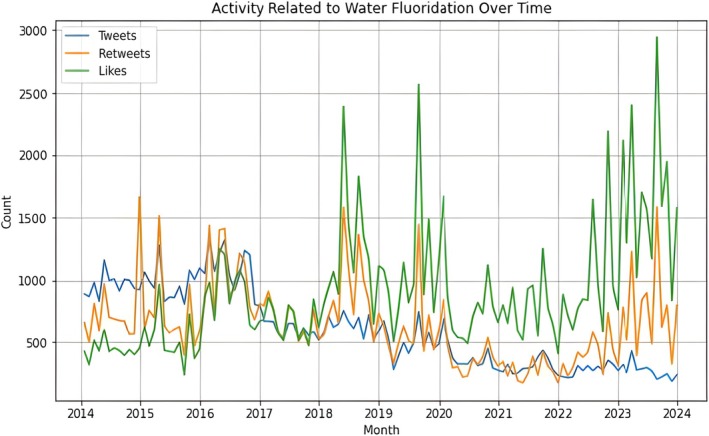
Community water fluoridation‐related activities on “X” (2014–2024). This figure shows the volume of tweets (blue), retweets (orange), and likes (green) over time. Peaks are observed in 2015–2016, followed by a general decline until 2022. However, engagement levels (retweets and likes) show increased fluctuations from 2019 onward, indicating significant variability in public interest. [Color figure can be viewed at wileyonlinelibrary.com]

**FIGURE 3:**
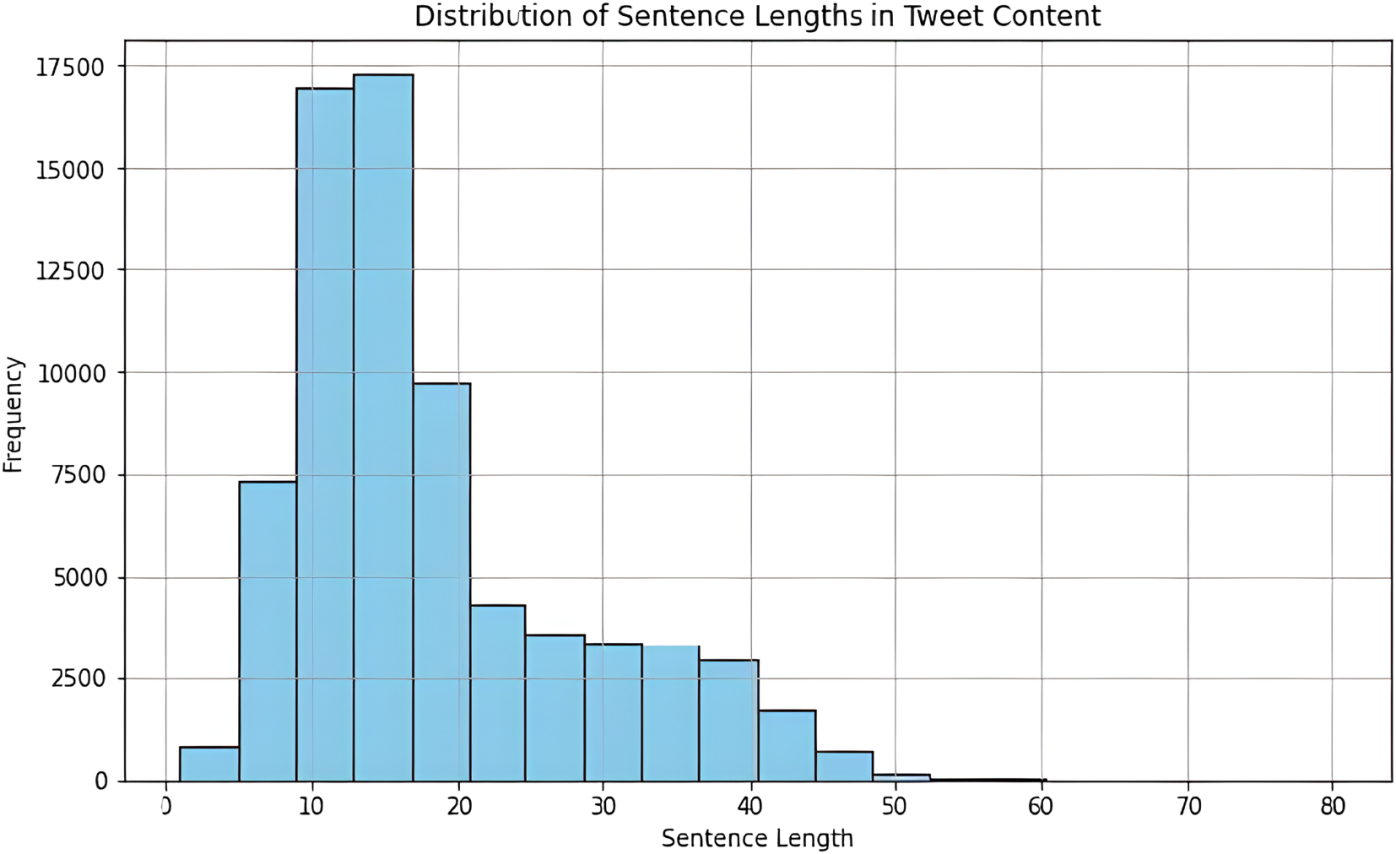
Distribution of sentence lengths in community water fluoridation tweets (2014–2024). This figure illustrates the distribution of tweet lengths (measured in characters), with the highest frequency occurring around 15 characters. The histogram shows a sharp decline as sentence length increases, highlighting the concise nature of discussions related to CWF on “X.” The long‐tail distribution suggests occasional longer tweets that provide more detailed discussions. [Color figure can be viewed at wileyonlinelibrary.com]

A total of 78,914 English‐language tweets related to CWF were extracted from January 2014 to December 2023, with 72,309 tweets finally analyzed after data processing. The analysis revealed a gradual decline in the proportion of tweets related to CWF, with minor fluctuations reaching a peak in 2016–2017 and a moderate and substantial decline following 2018. Despite the decrease in tweet generation, engagement activities, such as retweets and favorites, exhibited significant variability (Figure 2). This implies that, despite the topic's ongoing interest, engagement levels are primarily driven by specific tweets or events. In addition, most tweets had a length of 10 to 30 characters, with 15 characters being the most common tweet length. The frequency of tweets progressively decreases as the length of a sentence increases, with only a small number of tweets exceeding 80 characters (Figure 3).

### 3.2 Textual Sentiment Analyses

#### 3.2.1 Sentiment Trends and Pattern

**FIGURE 4:**
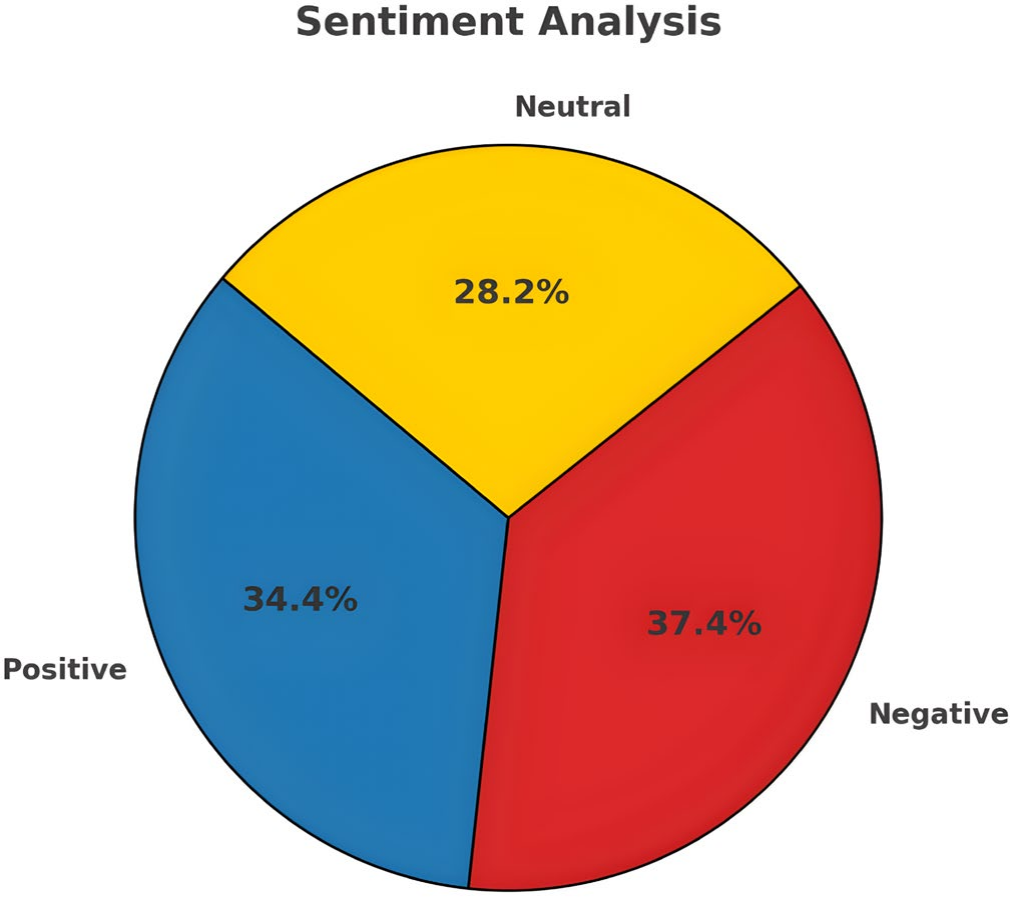
Overall sentiments about CWF on “X.” This figure presents the sentiment distribution in tweets about CWF. The analysis reveals that 37.4% of tweets express negative sentiments, 34.4% are positive, and 28.2% are neutral. This distribution highlights the polarized nature of public opinion on the topic, with a nearly equal balance of support and opposition. The presence of a significant neutral category suggests ongoing discourse and uncertainty regarding community water fluoridation. [Color figure can be viewed at wileyonlinelibrary.com]

**FIGURE 5:**
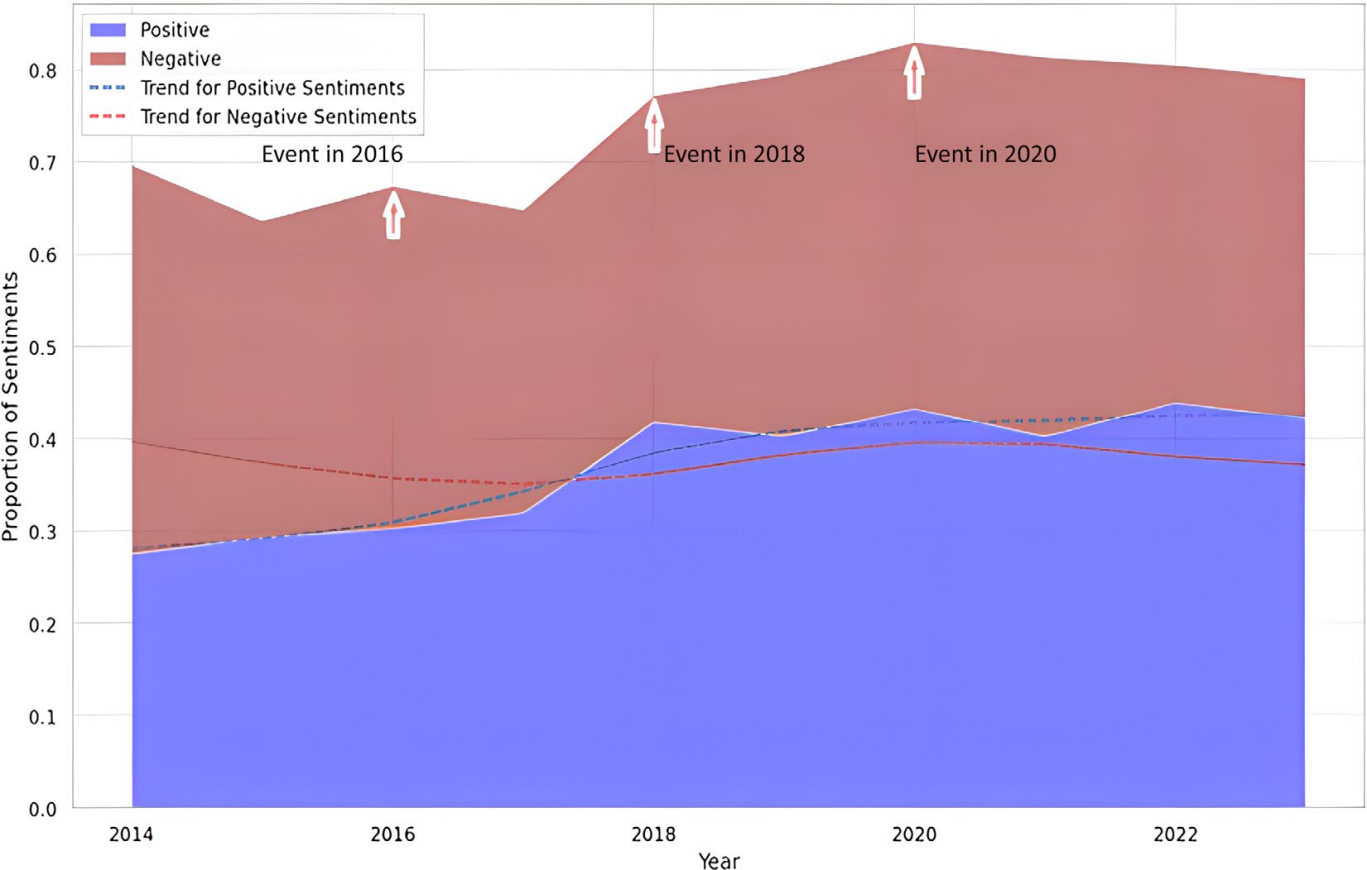
Sentiment trends about CWF‐related tweets over time. This figure illustrates the temporal changes in sentiment related to CWF on “X” from 2014 to 2023. The shaded regions indicate the proportion of positive (blue) and negative (red) sentiments over time, while trend lines show overall sentiment shifts. Notable spikes in sentiment coincide with key events in 2016, 2018, and 2020, likely driven by policy debates, scientific studies, or media coverage. The data suggest a gradual increase in positive sentiment, while negative sentiment remains prominent but fluctuates over time. [Color figure can be viewed at wileyonlinelibrary.com]

Of the tweets regarding CWF, 37.4% conveyed a negative emotion, 34.4% were positive, and 28.2% were neutral, indicating a relatively equal distribution of sentiments (Figure 4). Furthermore, reactions to water fluoridation‐related events are reflected in the proportional variation of positive and negative sentiments. In 2016, 2018, and 2020, substantial changes in public sentiments were observed, suggesting that these events in the years significantly impacted public opinion. These annotations indicated a strong correlation between sentiment trends and historical events, emphasizing the impact of societal events on public emotions (Figure 5).

#### 3.2.2 Word Sentiment Analysis

**FIGURE 6:**
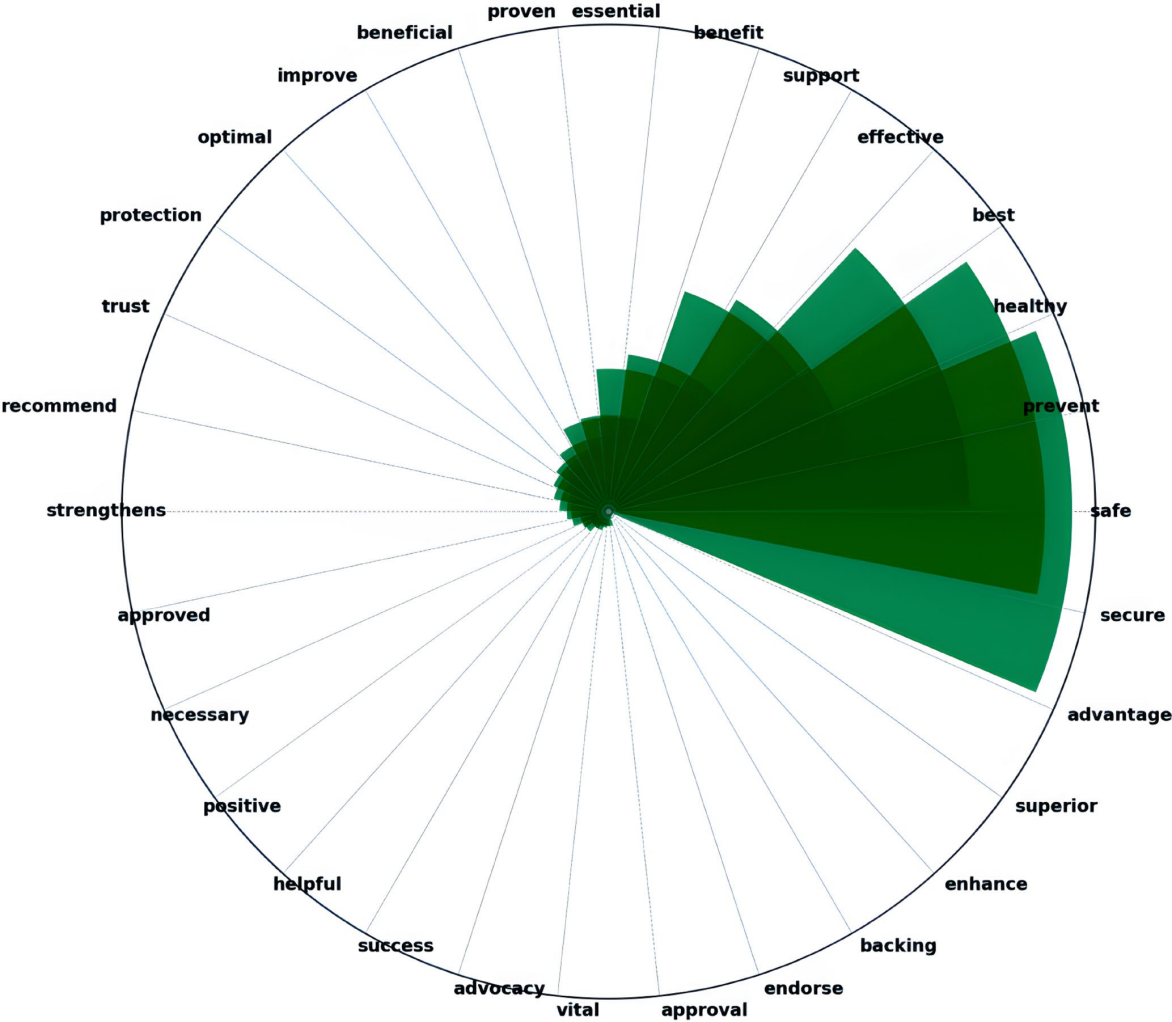
Top positive (favorable) words discussed in CWF‐related tweets. This figure highlights the most frequently used positive words in tweets related to community water fluoridation. Prominent terms such as “safe,” “prevent,” and “healthy” suggest a strong public perception of fluoridation as beneficial for oral health. Other terms like ‘trust,’ “support,” and “effective” indicate endorsement and confidence in fluoridation policies. The visualization underscores the positive language associated with discussions on the topic. [Color figure can be viewed at wileyonlinelibrary.com]

The presentation of the most favorable (positive) terms the users have used to describe CWF indicates its social acceptance. Words like “safe,” “prevent,” “healthy,” and “beneficial” were employed most frequently to underscore the health advantages of water fluoridation. Words like “essential” and “effective” emphasize its importance and efficiency. The users' high regard and trust for the scientific foundation of fluoridation are indicated by the frequency with which the terms “approve,” “trust,” and “recommend” were used (Figure 6).

**FIGURE 7:**
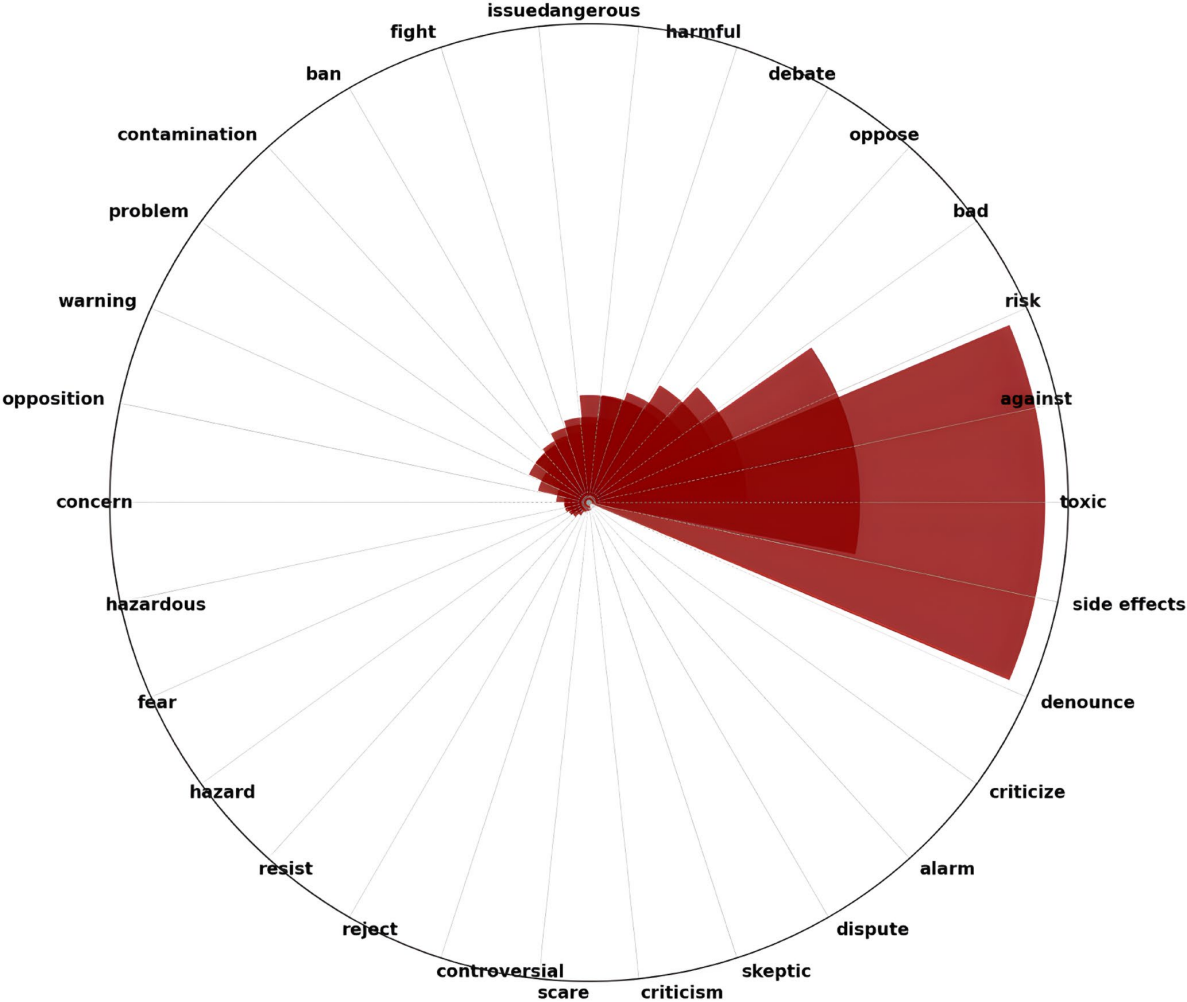
Top negative (unfavorable) words discussed in CWF‐related tweets. This figure highlights the most frequently used negative words in tweets related to community water fluoridation. Prominent terms such as “toxic,” “harmful,” and “dangerous” suggest ongoing public concerns about potential health risks. Other commonly occurring words like “risk,” “opposition,” and “side effects” indicate skepticism and resistance to fluoridation policies. The visualization underscores the language of caution and debate in discussions surrounding this public health measure. [Color figure can be viewed at wileyonlinelibrary.com]

In contrast, examining unfavorable (negative) terms illustrates divergent viewpoints. Concerns regarding the safety of water fluoridation are raised by the phrases “toxic,” “harmful,” and “dangerous.” The terms “risk,” “side effects,” and “controversial” all serve to emphasize the ongoing discourse concerning potential adverse effects. The utilization of the terms “oppose,” “fight,” and “ban” implies that there is a concerted effort to oppose fluoridation policies. The use of terms such as “dispute,” “criticize,” and “skeptic” suggests that the narratives that advocate for fluoridation have been approached with caution by some users (Figure 7).

#### 3.2.3 Word Co‐Occurrence Network Analysis

**FIGURE 8:**
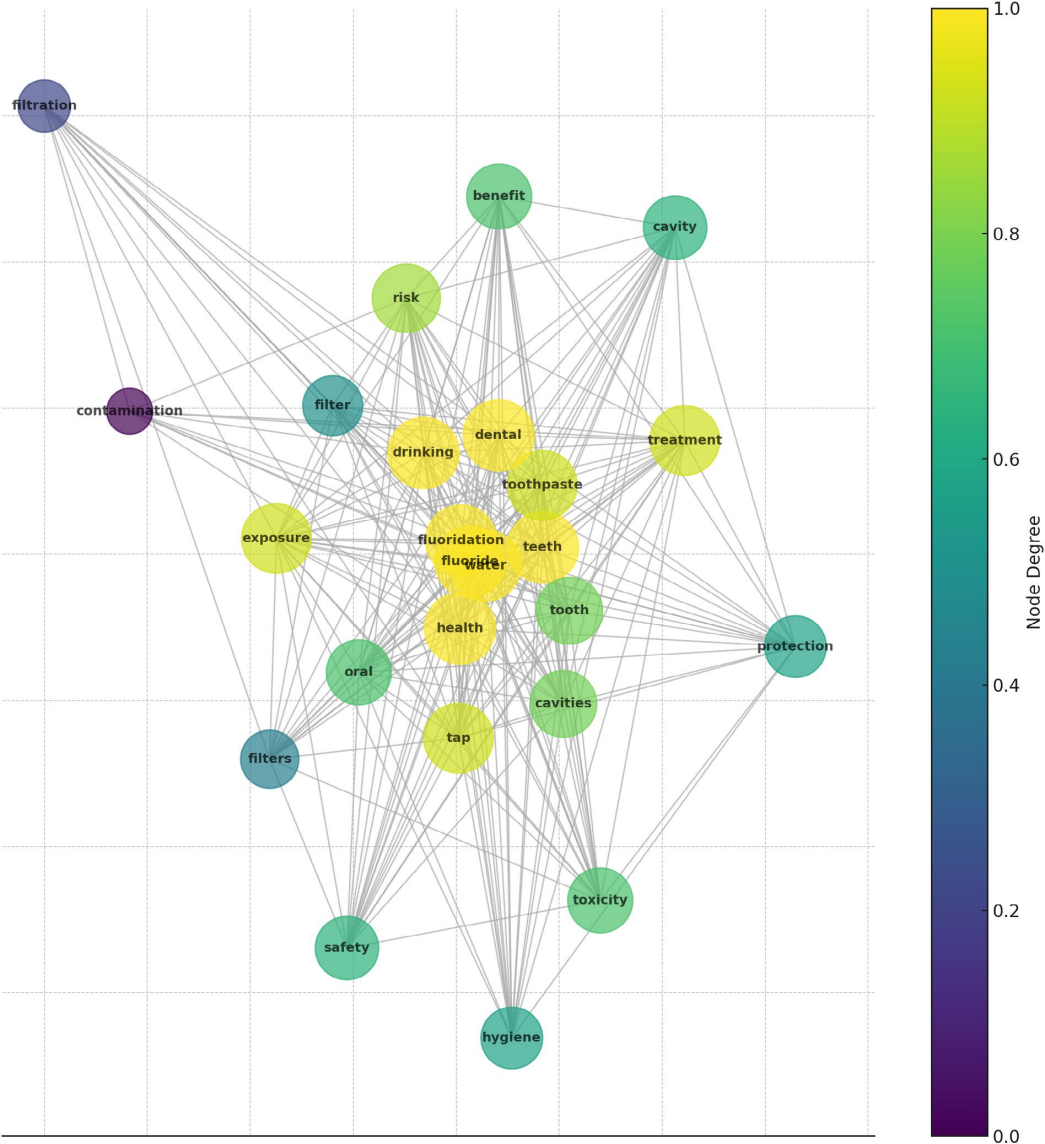
Word co‐occurrence network analysis of CWF discussions on “X.” This figure represents the word co‐occurrence network in CWF‐related discussions on “X.” The nodes represent frequently used words, and the edges indicate their co‐occurrence in tweets. Central terms such as “fluoridation,” “health,” and “teeth” suggest a focus on dental health, while “safety,” “toxicity,” and “contamination” reflect concerns about water quality. The color scale represents node degree, with high‐degree nodes indicating key discussion themes. [Color figure can be viewed at wileyonlinelibrary.com]

Word co‐occurrence network analysis investigated the relationships between keywords associated with water fluoridation in tweet discussions. The primary focus of the debate was on central hubs, including “fluoride,” “water,” and “health,” which have high node degrees, indicating they were highly connected to other keywords. “Safety issues” (including ‘risk,’ “safety,” and “toxicity”) and “dental health” (with phrases such as “teeth,” “dental,” and “toothpaste”) were notable clusters that indicated specific debate topics. “Infection” “filters,” and “filtration” indicated concerns regarding implementing safety measures and water purity. The correlations between “benefit,” “protection,” and “treatment” suggested the necessity of educational and lobbying initiatives (Figure 8).

#### 3.2.4 Topic Modeling and Issue Tweet Analysis

**FIGURE 9:**
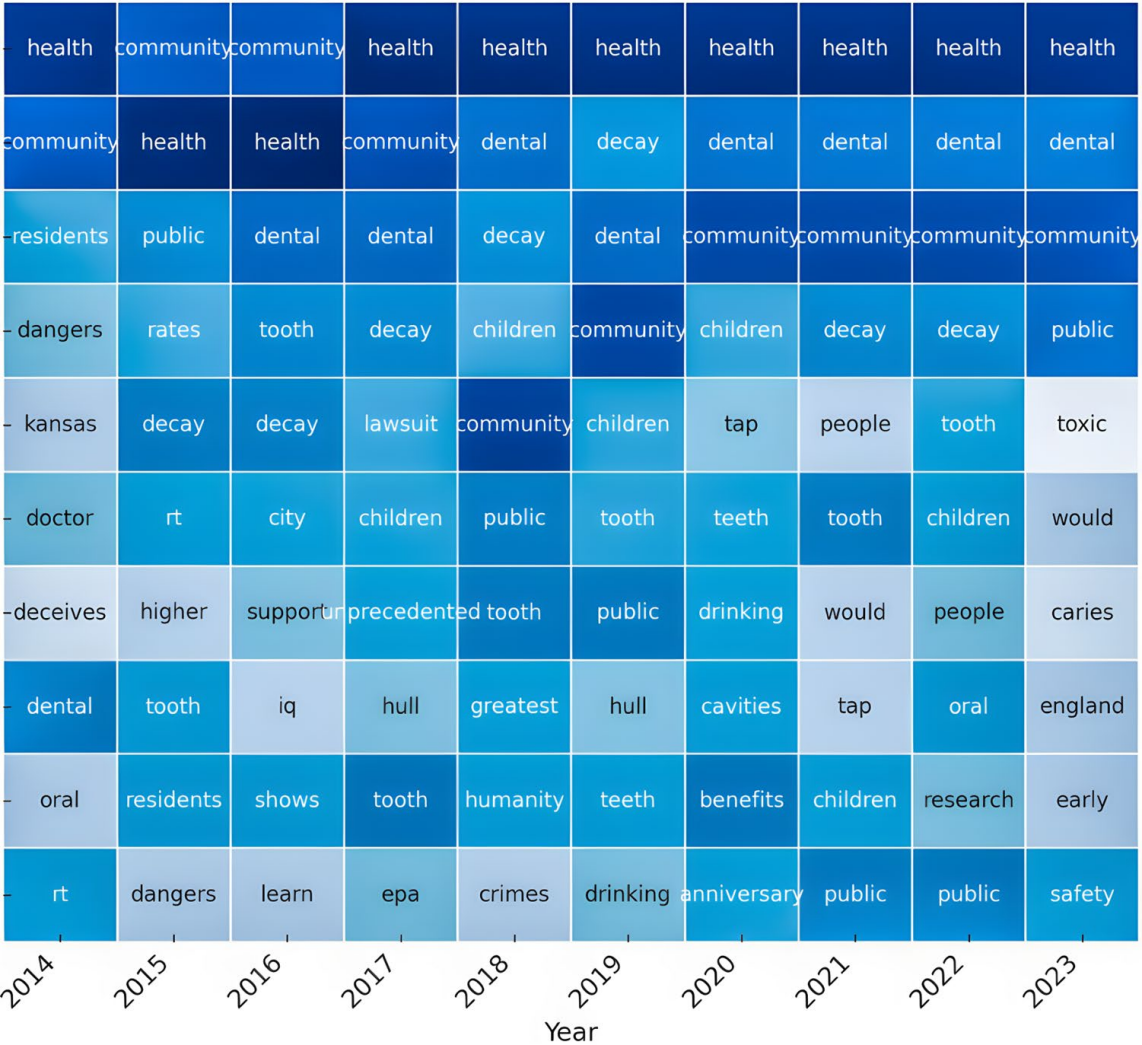
Topic modeling and issue tweet analysis of CWF‐related discussions on “X.” This figure presents the results of topic modeling on tweets about community water fluoridation (CWF) over time. The color intensity represents word frequency, with darker shades indicating higher frequencies. Key recurring topics include health effects, community acceptance, and children's dental health. Notable terms such as “health,” “decay,” “public,” and “tooth” highlight persistent public discourse on the benefits and concerns related to CWF. The analysis captures how discussions evolved, reflecting shifting priorities and emerging debates. [Color figure can be viewed at wileyonlinelibrary.com]

The recurring topics in “X” conversations on CWF are illustrated in the tabulated list of prominent terms from 2014 to 2023 (Figure 9). The persistent concerns regarding the health effects and community acceptance are indicated by the consistent periodic recurrence of critical issues, including “health,” “community,” “dental,” and “decay.” The term “children” has frequently been used for years, suggesting concerns regarding children's dental health. In 2023, the terms “toxic” and “safety” were used to underscore periods of elevated apprehension regarding the potential risks of fluoridation. In addition, specific years exhibit a concentration on subjects such as “benefits,” “research,” and “cavities,” suggesting a trend toward evaluating the scientific credibility and efficacy of fluoridation methods. The variations in public discourse regarding CWF over the years are underscored by these patterns.

#### 3.2.5 Model Performance Evaluation

Table 1 summarizes the performance evaluation of various sentiment analysis models. The highest accuracy (88.8%) and AUC value (0.95) were exhibited by the Logistic Regression model, indicating its exceptional performance in accurately classifying sentiments. Naive Bayes exhibited promising results, obtaining an AUC value of 0.85 and an accuracy rate of 78.6%. The Random Forest algorithm exhibited a high AUC value of 0.90 and a similar level of accuracy at 77.3%, indicating its high and reliable performance. The Decision Tree model showed the lowest performance, with an AUC value of 0.75 and an accuracy of 77%. The results of this study suggest that the model is less reliable than the others (Table 1).

**TABLE 1:** Model performance and evaluation details.

Model	Accuracy (%)	Precision (%)	Recall (%)	F1 score (%)	AUC value
Logistic regression	88.83	88.87	88.83	88.82	0.95
Decision tree	77.09	77.14	77.09	77.07	0.75
Naive bayes	78.63	78.79	78.63	78.59	0.85
Random forest	77.31	78.80	77.31	77.12	0.90

*Note*: This table summarizes the performance metrics of various sentiment analysis models used in the study. Metrics such as accuracy, precision, recall, F1 score, and area under the curve (AUC) are provided to evaluate each model's effectiveness in classifying sentiments related to CWF discussions on “X.”

## 4 Discussion

Our sentiment analysis of community water fluoridation (CWF) on “X” over the past decade revealed a detailed understanding of public opinion. The study showed that 37.4% of tweets expressed negative sentiments toward CWF, compared to 34.4% positive and 28.2% neutral sentiments. The slight disparity between positive and negative attitudes suggests the subject is polarizing, with public opinion almost equally divided (Data S2).

We found significant variations in public involvement in CWF discussions, with surges in 2015–2016 and a more marked decrease after 2018. The data indicates that the public's interest in CWF has slowly diminished, possibly overshadowed by other health‐related debates. An investigation on media coverage of health matters revealed that health subjects frequently vie for attention, with more dramatic or urgent issues typically eclipsing others [22]. This phenomenon is particularly evident in talks around digital health, where prominent subjects such as COVID‐19 and mental health have overwhelmingly dominated public conversations on social media platforms, reducing focus on topics like CWF [23]. Nevertheless, specific tweets or occurrences continue to generate substantial interaction, highlighting the significance of context in debates on social media.

We found that the higher spikes in negative sentiment often corresponded with significant CWF‐related events. For example, in 2015, the U.S. Department of Health and Human Services updated its guidelines for optimal fluoride levels in drinking water, leading to a surge in tweets discussing the changes and their implications [24]. Similarly, in May 2013 and May 2016, the rejection of a proposal to add fluoride to the drinking water in Portland, Oregon, and Albuquerque, New Mexico, sparked a significant increase in Twitter activity [25].

Our findings align with previous research by Oh et al. [11], which reported a significant proportion of negative sentiments towards water fluoridation on Twitter. Furthermore, Mertz and Allukian [5, 6] highlighted the role of mis(dis)information in amplifying negative sentiments online. Rodrigues et al. [26] also found that these negative sentiments towards health interventions are more likely to become polarized, shared, and retweeted, contributing to mis(dis)information.

Our research emphasizes the importance of analyzing neutral sentiments, which can swing toward either positive or negative based on new information. Neutral perceptions, comprising 28.2% of the sentiments, represent a significant portion of public opinion. While we did not analyze this subset to determine whether it reflects ambivalence, lack of knowledge, or another sentiment type, this represents an area for future investigation. Understanding the underlying drivers of neutrality could provide deeper insights into public perceptions of CWF. For instance, neutral sentiments might indicate a lack of awareness or engagement with the topic, reflecting opportunities for targeted educational campaigns. Johnson et al. [25] highlighted that neutral opinions could shift dramatically with new information, potentially altering the balance of online views and influencing public discourse. Monitoring these neutral sentiments is essential for anticipating and managing changes in public opinion on CWF.

Our word sentiment analysis identified key positive terms like “safe,” “prevent,” and “healthy,” indicating support for the health benefits of CWF. Conversely, negative terms such as “toxic,” “harmful,” and “dangerous” reflect ongoing safety concerns. Similar to our results, Oh et al. observed that negative terms like “poison” and “waste” were frequently used, reflecting significant public concern over the safety and potential risks of fluoridation [11]. This consistency suggests that public skepticism toward CWF remains persistent despite varying methodologies and timeframes.

The co‐occurrence network analysis highlighted central themes, particularly clusters around “safety issues” and “dental health.” This is consistent with findings by Mackert et al. [12], who observed that concerns about safety and potential side effects were prevalent in social media discussions on dental health interventions. Additionally, topic modeling revealed ongoing concerns about health effects and community acceptance of CWF, with significant attention to children's dental health. We found that in recent years, there has been sustained apprehension about potential risks associated with CWF, which aligns with findings by Oh et al. [11], noting that safety and health impacts were dominant themes in Twitter discussions. Furthermore, Kanchan and Gaidhane [23] found that the perceived credibility of sources and the emotional tone of messages significantly impacted public engagement and sentiment.

Performance evaluation is a critical parameter for analyzing the accuracy of various sentiment analysis models. In our study, we selected Logistic Regression, Naive Bayes, Decision Tree, and Random Forest due to their proven effectiveness in text classification. Logistic Regression was chosen for its simplicity, interpretability, and ability to capture linear relationships in high‐dimensional data [27, 28]. Naive Bayes is efficient for handling sparse data in natural language processing, despite its simplifying assumption of feature independence [29]. Random Forest's ensemble approach reduces overfitting and effectively captures non‐linear relationships [30]. In contrast, Decision Trees, though interpretable, are more prone to overfitting in high‐dimensional contexts [31]. Overall, our experimental results showed that Logistic Regression outperformed the other models, aligning with previous research. This selection reinforces the relevance of these models in addressing sentiment analysis challenges.

Additionally, the recent Cochrane systematic review on water fluoridation (2024) highlights that while CWF remains effective in reducing dental caries, the magnitude of its effect may be lower than earlier estimates, in part due to additional fluoride sources now widely available [28]. Importantly, this review underscores the equity debate, noting that communities with less access to alternative fluoride interventions may still benefit substantially from CWF. This resonates with our findings, where neutral sentiments—representing a potential “swing” group—could be influenced by targeted messaging about both the protective and equitable aspects of fluoridation policies. Policymakers should thus consider not only the efficacy but also the fairness and reach of CWF initiatives when designing public health strategies.

Given our key finding that public sentiment towards CWF shows a nearly equal distribution of negative, positive, and neutral sentiments, we recommend developing comprehensive monitoring systems and strategies to manage public sentiment about CWF effectively. Engaging with the public, communicating transparently with health authorities, and using credible influencers can help balance the discussion. Real‐time sentiment analysis and tracking key accounts can quickly spot and address new trends, reducing mis(dis)information and limiting negative sentiments from spreading [23, 26]. Additionally, developing reliable fact‐checking systems specifically for CWF concerns is crucial. These systems should educate the public, address their concerns, and use automated tools to flag mis(dis)information. Educational campaigns that explain the benefits and safety of CWF, backed by scientific evidence, can help clear up misconceptions and build trust [11, 26].

This study has several limitations that warrant consideration. First, temporal and demographic biases may have influenced the dataset. Changes in Twitter's user demographics over the years, as well as region‐specific events, could have impacted the observed sentiment trends. While these biases were not explicitly controlled for, we addressed them partially by contextualizing significant sentiment spikes and linking them to major events, such as policy debates and media coverage. Future studies should integrate demographic and geographic metadata to mitigate these biases and enhance the precision of analytical insights. Second, the analysis was limited to English‐language tweets, potentially excluding important discussions in other languages. This limitation may restrict the generalisability of the findings to non‐English‐speaking populations. Additionally, while automated sentiment analysis tools are efficient and effective for processing large datasets, they may not fully capture the nuance of human emotions and context. Incorporating manual validation alongside automated tools in future studies could improve the accuracy and reliability of sentiment classification. Third, the study's timeframe is a potential limitation. Social media trends evolve rapidly, and analyzing a static time period may not fully capture the dynamic nature of public discourse. Expanding the study period or conducting longitudinal studies would provide deeper insights into how sentiments change over time and help identify emerging trends. Finally, this study focused solely on a single platform, “X” (formerly Twitter). While “X” provides valuable insights, expanding the analysis to include other social media platforms, such as Facebook and Instagram, would offer a more comprehensive view of public sentiment. Different platforms attract diverse user demographics and may host varied discussions on CWF. We recommend that future research explore multiple platforms to capture a broader spectrum of public opinion and enhance the generalizability of findings.

*Moreover, while we examined tweet content and engagement, we did not investigate the profiles or credibility of the users posting these tweets—whether they were general users, key opinion leaders, bots, or institutional accounts. Distinguishing these user types and assessing their credibility could yield valuable scientometric insights, particularly regarding how polarizing narratives originate or are amplified. As our primary objective was to explore overall sentiment and topical trends surrounding CWF, future studies should integrate user‐level analyses to better understand and address mis(dis)information*.

Overall, the study highlights the evolving nature of public sentiment about CWF on “X” and emphasizes the importance of real‐time monitoring to guide public health strategies. By using machine learning to analyze social media data, this research provides actionable insights for improving health communication and policymaking in today's digital age. Practically, the findings suggest that public health policymakers should develop targeted interventions to address misinformation and engage neutral audiences. Strategies could include creating localized, culturally relevant educational campaigns and leveraging trusted community influencers to amplify accurate information. Establishing partnerships with social media platforms to monitor and flag misleading content is another actionable step. These efforts can help build trust in CWF and ensure informed decision‐making within communities. While this study offers valuable information, more research on CWF‐related sentiments across various social media platforms is needed to ensure a holistic understanding and more effective public health strategies.

## Supporting information


**Data S1.** Supporting Information.

**Data S2.** Supporting Information.

## Data Availability

The data that support the findings of this study are available on request from the corresponding author. The data are not publicly available due to privacy or ethical restrictions.
