# Supplementary material for: Mapping the “X” Debate: Water Fluoridation Sentiment Analysis With Advanced Machine Learning
Source: J Public Health Dent. 2025 May 7;85(3):231–43. doi: 10.1111/jphd.12669 (PMC12418723; doi:10.1111/jphd.12669)
Supplement: Supplementary file 1 — Data S1. Supporting Information. [file JPHD-85-231-s001.docx]

**Supplementary File 1:**

**Sample of Tweets showing positive, negative and neutral emotions about CWF on ‘X’.**

| Opinion | Tweet | Sentiment Polarity Score (Range +1 to -1) |
| --- | --- | --- |
| Positive | "Absolutely love how our community is protecting our teeth by fluoridating the water. It's a no-brainer for better dental health! 🦷💧 #HealthFirst" | +0.9 |
| Positive | "Fluoridation in water is a win for public health! It's reassuring to know our kids are getting extra protection. 💪✨ #HealthySmiles" | +0.8 |
| Positive | "Finally, a decision to fluoridate our water! This is a huge step toward reducing cavities and improving overall dental health. Proud of my city! 😁 #OralHealth" | +0.85 |
| Positive | "Grateful for water fluoridation! Knowing it's helping prevent tooth decay for everyone, especially those who can't afford dental care, is just awesome. 🙌 #CommunityHealth" | +0.75 |
| Positive | "Fluoridated water has been a game-changer for my family’s dental health. Fewer cavities, stronger teeth! Thanks, science! 🧪🌟 #FluorideWorks" | +0.8 |
| Positive | "I’ve seen firsthand how fluoridated water has helped reduce cavities in kids where I work. It’s an essential public health measure. 👏 #PublicHealth" | +0.7 |
| Positive | "Thrilled that our town is keeping fluoridation in the water. It’s such a simple way to help so many. 😊 #DentalCare" | +0.75 |
| Positive | "When people complain about water fluoridation, I can’t help but wonder if they realize how much it helps with dental care. Super grateful for it! 🦷💙 #HealthyCommunities" | +0.65 |
| Positive | "Fluoridation is a proven method to improve dental health. I’m so glad our city supports it. More smiles, less decay! 😄 #SupportFluoride" | +0.7 |
| Positive | "If you care about public health, you should care about fluoridation. It’s made a massive difference in reducing tooth decay. Proud to support this! 🏥💧 #OralHealthMatters" | +0.9 |
| Negative | "I can’t believe they’re still pushing water fluoridation. It feels like we’re being forced to ingest chemicals without our consent. 😠 #FluorideFree" | -0.8 |
| Negative | "Adding fluoride to our water is just another way the government tries to control us. I don’t trust it, and I don’t want it. 😤 #SayNoToFluoride" | -0.85 |
| Negative | "Why are we still using fluoride in our water? It’s outdated and unnecessary. We deserve the choice not to consume it. 😒 #FluorideFree" | -0.7 |
| Negative | "Fluoride in water? No thanks! I don’t need the government deciding what’s best for my health. Feels like a violation of my rights. 🚫 #StopFluoridation" | -0.75 |
| Negative | "Water fluoridation is just mass medication without consent. It’s wrong on so many levels, and I’m furious that it's still happening. 😡 #FluorideIsToxic" | -0.9 |
| Negative | "It’s crazy how people just accept water fluoridation like it’s normal. It’s not! We shouldn’t be forced to consume chemicals. 😠 #AntiFluoride" | -0.7 |
| Negative | "I’m deeply concerned about the long-term effects of fluoride in our water. Why are we being treated like lab rats? 🧪 #FluorideFreeFuture" | -0.8 |
| Negative | "Fluoride in the water is nothing but a big experiment on the public. We deserve better than this! 😡 #NoToFluoride" | -0.85 |
| Negative | "I feel betrayed knowing our water is fluoridated without proper public consultation. It’s not right. 😞 #FluorideFree" | -0.75 |
| Negative | "Fluoride in water is dangerous and unnecessary. We need to put a stop to this practice now before it’s too late. ⚠️ #FluorideIsPoison" | -0.9 |
| Neutral | "Fluoridation has been around for years, but I’m still not sure what to think about it. There are pros and cons to both sides. 🤔 #OnTheFence" | 0.0 |
| Neutral | "Honestly, I don’t have a strong opinion on water fluoridation. I guess it’s good for some, but I see why others might oppose it. 🤷‍♂️ #NeutralThoughts" | 0.0 |
| Neutral | "I’ve heard a lot about water fluoridation, but I’m still undecided. Both sides make valid points. 🤨 #FluorideDebate" | 0.0 |
| Neutral | "Not sure what to believe about fluoride in water. Seems like everyone has a different opinion, and it’s hard to know who’s right. 😕 #Confused" | 0.0 |
| Neutral | "Water fluoridation isn’t something I think about often. It’s just part of life, I guess. 🤷‍♀️ #Whatever" | 0.0 |
| Neutral | "There’s a lot of debate about water fluoridation, but I’m still on the fence. It’s hard to say what’s really best. 😐 #FluorideDiscussion" | 0.0 |
| Neutral | "I don’t really have a stance on fluoridation. I can see both sides, so I’m just staying neutral. 🤷 #NoStrongFeelings" | 0.0 |
| Neutral | "I’ve read arguments for and against water fluoridation. Honestly, I’m not convinced either way. Just seems like another debate. 😬 #Undecided" | 0.0 |
| Neutral | "It’s interesting to see how divided people are over water fluoridation. I don’t have a strong opinion myself. 🤔 #FluorideTalk" | 0.0 |
| Neutral | "Water fluoridation is one of those things I just don’t think about much. People seem passionate, though. 🤷‍♂️ #Neutral" | 0.0 |
