# Supplementary material for: Mapping the “X” Debate: Water Fluoridation Sentiment Analysis With Advanced Machine Learning
Source: J Public Health Dent. 2025 May 7;85(3):231–43. doi: 10.1111/jphd.12669 (PMC12418723; doi:10.1111/jphd.12669)
Supplement: Supplementary file 2 — Data S2. Supporting Information. [file JPHD-85-231-s002.docx]

| **Category** | **Details** |
| --- | --- |
| **Engagement Metrics** | 78,914 tweets extracted; 72,309 tweets analysed. Decline in tweet volume post-2018 with engagement variability. Most tweets between 10-30 characters. |
| **Sentiment Percentages** | Positive: 34.4%, Negative: 37.4%, Neutral: 28.2%. Variations in sentiment trends linked to historical events in 2016, 2018, and 2020. |
| **Top Positive Keywords** | Safe, Prevent, Healthy, Beneficial, Essential, Effective, Approve, Trust, Recommend. |
| **Top Negative Keywords** | Toxic, Harmful, Dangerous, Risk, Side Effects, Controversial, Oppose, Fight, Ban. |
| **Keyword Co-occurrence Clusters** | Safety Issues: risk, safety, toxicity; Dental Health: teeth, dental, toothpaste; Water Purity: infection, filters, filtration. |
| **Tweet Length Analysis** | Most tweets were 10-30 characters; tweet frequency decreases as length increases, with very few tweets exceeding 80 characters. |
| **Sentiment Trends Over Time** | Significant sentiment changes observed during key years (2016, 2018, 2020), likely linked to historical events. |
| **Topic Modelling** | Recurring topics: Health, Community, Dental, Decay, Children. Notable terms in specific years include 'toxic' and 'safety' (2023). |
| **Model Performance** | Logistic Regression achieved highest accuracy (88.8%) and AUC (0.95). Naive Bayes and Random Forest showed reliable results, while Decision Tree underperformed. |

**Supplementary File 2-** **Comprehensive summary of engagement metrics, sentiment analysis, keyword clusters, tweet characteristics, topic modelling insights, and model performance in Community Water Fluoridation (CWF) discussions on 'X' (2014–2023).**
